# Supplementary material for: The Systems Biology Research Tool: evolvable open-source software
Source: BMC Syst Biol. 2008 Jun 29;2:55. doi: 10.1186/1752-0509-2-55 (PMC2446383; doi:10.1186/1752-0509-2-55)
Supplement: Additional file 1 — SBRT Archive. An archive of the current version of the Systems Biology Research Tool. [file 1752-0509-2-55-S1.zip › sbrt-1.4.0/doc/developers_guide/api/sbrt/shell/io/WorkingDirectory.html]

WorkingDirectory


|  |  |  |
| --- | --- | --- |
| **Overview** \| **Package** \| **Class** \| **Use** \| **Tree** \| **Deprecated** \| **Index** \| **Help** | |  |
| **PREV CLASS**   **NEXT CLASS** | **FRAMES**    **NO FRAMES**     **All Classes** |  |
| SUMMARY: NESTED \| FIELD \| CONSTR \| METHOD | DETAIL: FIELD \| CONSTR \| METHOD |  |


---


## sbrt.shell.io Class WorkingDirectory

```
java.lang.Object
  sbrt.shell.io.WorkingDirectory
```

---

``` public class WorkingDirectory extends java.lang.Object ```

This class is used to manage the working directory of the SBRT.

**Author:**
:   This class was written and documented by
    Jeremiah Wright while in the Wagner lab.

---

**Constructor Summary**

`WorkingDirectory()`


| **Method Summary** | |
| --- | --- |
| `static java.io.File` | `get()`             Returns the current working directory. |
| `static void` | `set(java.io.File workingDir)`             Sets the working directory to the provided value. |
| `static void` | `set(java.lang.String workingDir)`             Sets the working directory to the provided value. |

**Methods inherited from class java.lang.Object**

`clone, equals, finalize, getClass, hashCode, notify, notifyAll, toString, wait, wait, wait`

**Constructor Detail**

### WorkingDirectory

```
public WorkingDirectory()
```


**Method Detail**

### get

```
public static java.io.File get()
```

:   Returns the current working directory.

    :   **Returns:**: the current working directory.

---


### set

```
public static void set(java.io.File workingDir)
```

:   Sets the working directory to the provided value.

    :   **Parameters:**: `workingDir` - the new working directory. **Throws:**: `ApplicationException` - if the provided file is not a directory.

---


### set

```
public static void set(java.lang.String workingDir)
```

:   Sets the working directory to the provided value.

    :   **Parameters:**: `workingDir` - the name of the new working directory. **Throws:**: `ApplicationException` - if the specified file is not a directory.


---


|  |  |  |
| --- | --- | --- |
| **Overview** \| **Package** \| **Class** \| **Use** \| **Tree** \| **Deprecated** \| **Index** \| **Help** | |  |
| **PREV CLASS**   **NEXT CLASS** | **FRAMES**    **NO FRAMES**     **All Classes** |  |
| SUMMARY: NESTED \| FIELD \| CONSTR \| METHOD | DETAIL: FIELD \| CONSTR \| METHOD |  |


---
